# Targeting TRAF3 Downstream Signaling Pathways in B cell Neoplasms

**DOI:** 10.4172/1948-5956.1000327

**Published:** 2015-02-25

**Authors:** Carissa R Moore, Shanique KE Edwards, Ping Xie

**Affiliations:** 1 Department of Cell Biology and Neuroscience, New Jersey, USA; 2 Graduate Program in Molecular Biosciences, Rutgers University, Piscataway, New Jersey, USA; 3 Member, Rutgers Cancer Institute of New Jersey, USA

**Keywords:** TRAF3, B lymphoma, Multiple myeloma, NF-κB2, AD 198, MCC, Sox5

## Abstract

B cell neoplasms comprise >50% of blood cancers. However, many types of B cell malignancies remain incurable. Identification and validation of novel genetic risk factors and oncogenic signaling pathways are imperative for the development of new therapeutic strategies. We and others recently identified TRAF3, a cytoplasmic adaptor protein, as a novel tumor suppressor in B lymphocytes. We found that TRAF3 inactivation results in prolonged survival of mature B cells, which eventually leads to spontaneous development of B lymphomas in mice. Corroborating our findings, TRAF3 deletions and inactivating mutations frequently occur in human B cell chronic lymphocytic leukemia, splenic marginal zone lymphoma, mantle cell lymphoma, multiple myeloma, Waldenström’s macroglobulinemia, and Hodgkin lymphoma. In this context, we have been investigating TRAF3 signaling mechanisms in B cells, and are developing new therapeutic strategies to target TRAF3 downstream signaling pathways in B cell neoplasms. Here we discuss our new translational data that demonstrate the therapeutic potential of targeting TRAF3 downstream signaling pathways in B lymphoma and multiple myeloma.

## TRAF3: A New Tumor Suppressor Gene in B Lymphocytes

B cell neoplasms comprise >50% of blood cancers. Despite recent advances in treatment, many types of human B cell neoplasms remain incurable, highlighting a clear need for new therapeutic strategies [1–3]. Identification and validation of new genetic risk factors and critical oncogenic pathways are imperative for a better understanding of B cell malignant transformation and for the development of new therapeutic strategies [4–6]. Recent studies from our laboratory and others have identified TRAF3, a crucial regulator of B cell survival, as a novel tumor suppressor in B lymphocytes [7–17].

TRAF3 is a member of the tumor necrosis factor receptor (TNF-R)-associated factor (TRAF) family (TRAF1–7) of cytoplasmic adaptor proteins [18]. TRAF3 is used for signaling by receptors of the TNF-R superfamily, pattern recognition receptors (PRRs), and a number of viral proteins such as the Epstein-Barr virus encoded oncoprotein LMP1 [18]. Consistent with the shared usage of TRAF3 by so many receptors, mice made genetically deficient in TRAF3 exhibit global defects and die by 10 days after birth [19]. To circumvent experimental limitations imposed by the early mortality of TRAF3^−/−^ mice and, more specifically, to explore the *in vivo* functions of TRAF3 in B lymphocytes of adult mice, we recently generated and characterized B cell-specific TRAF3-deficient (B-TRAF3^−/−^; TRAF3^flox/flox^CD19^+/cre^) mice [7,8]. We found that specific deletion of TRAF3 in B lymphocytes results in marked peripheral B cell hyperplasia, due to remarkably prolonged survival of mature B cells independent of the B cell survival factor BAFF. This eventually leads to spontaneous development of splenic marginal zone lymphomas (MZL) or B1 lymphomas in mice by 18 months of age [7,8]. B lymphomas spontaneously developed in B-TRAF3^−/−^ mice are easily transplantable to immunodeficient NOD SCID recipient mice, demonstrating their malignant nature [8]. Corroborating our findings, biallelic deletions and inactivating mutations of the *Traf3* gene have been identified in human patients with a variety of B cell neoplasms, including multiple myeloma (MM), splenic marginal zone lymphoma (MZL), B cell chronic lymphocytic leukemia (B-CLL), mantle cell lymphoma (MCL), Waldenström’s macroglobulinemia (WM), and Hodgkin lymphoma [10–17]. Taken together, the findings obtained from both the B-TRAF3^−/−^ mice and human patients provide conclusive evidence that *Traf3* is a tumor suppressor gene in B lymphocytes.

## TRAF3 Downstream Signaling Pathways

**Figure 1:**
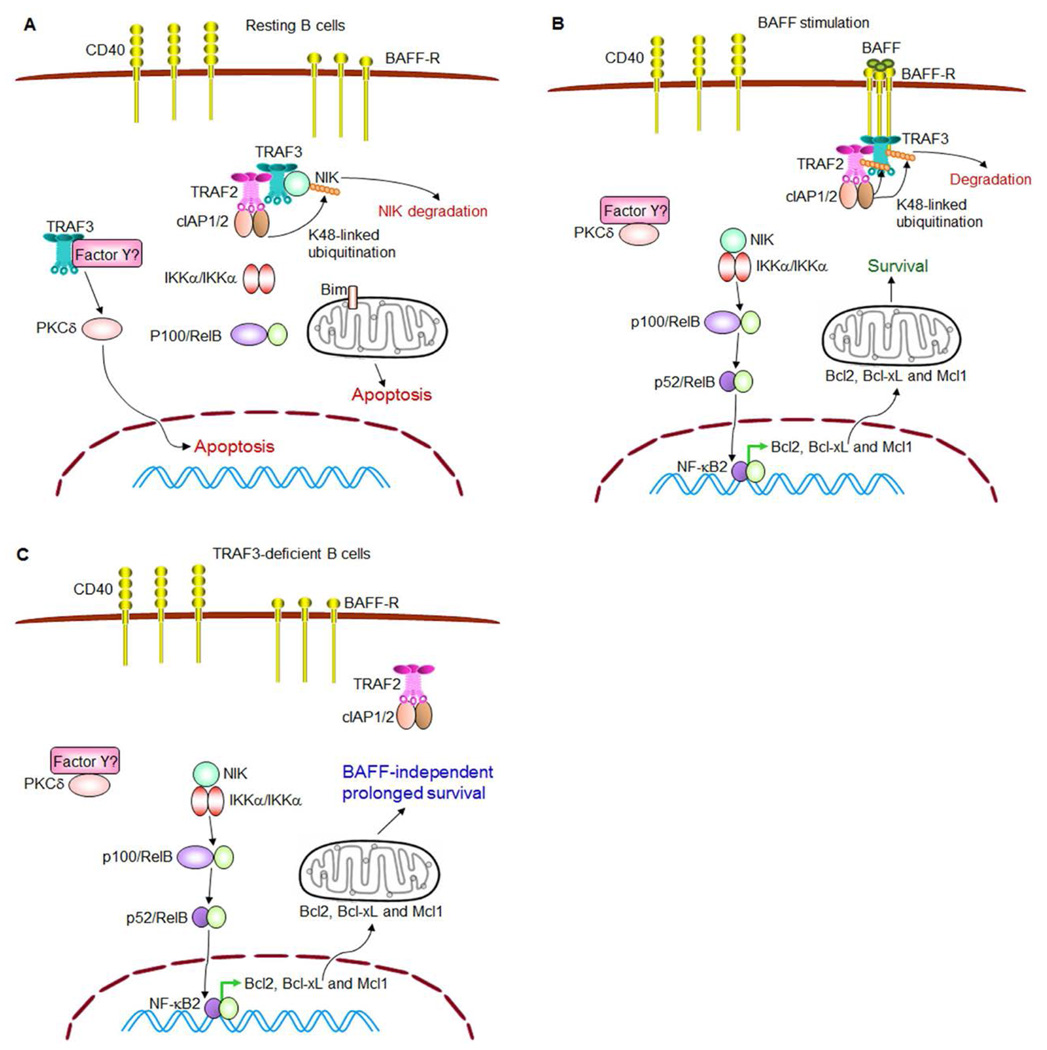
TRAF3 and BAFF signaling pathways in regulating B cell survival (**A**) TRAF3 promotes apoptosis in resting B cells. In the absence of stimulation, TRAF3 inhibits NF-κB2 activation while facilitating PKCδ nuclear translocation to promote B cell apoptosis. TRAF3 and TRAF2 constitutively form a complex with cIAP1/2 and NIK, targeting NIK for K48-linked polyubiquitination and degradation, thereby inhibiting NF-κB2 activation in B cells. How TRAF3 facilitates PKCδ nuclear translocation remains to be determined (depicted as through binding to an unknown protein or multi-protein complex Factor Y in the figure). (**B**) BAFF stimulates B cell survival. Upon ligand engagement, trimerized BAFF-R recruits TRAF3 and the associated TRAF2-cIAP1/2 complex to membrane rafts, and thus releases NIK, allowing NIK accumulation and NF-κB2 activation. Meanwhile, Factor Y is also released from TRAF3 and may sequester PKCδ in the cytosol. NF-κB2 activation together with reduced nuclear level of PKCδ functions to induce the expression of anti-apoptotic proteins, and thus promotes B cell survival. (**C**) TRAF3 deficiency causes BAFF-independent B cell survival. Similar to BAFF stimulation, deletion of TRAF3 from B cells (caused by either biallelic deletions or inactivating mutations of the *Traf3* gene) also releases NIK from the TRAF2-cIAP1/2 complex, causing constitutive NF-κB2 activation. In the absence of TRAF3, Factor Y may also sequester PKCδ in the cytosol. Therefore, constitutive NF-κB2 activation together with reduced nuclear level of PKCδ leads to BAFF-independent, prolonged survival of TRAF3^−/−^ B cells.

In pursuing the signaling pathways downstream of TRAF3 inactivation, we found that both TRAF3^−/−^ premalignant B cells and B lymphomas exhibit constitutive NF-κB2 activation but reduced PKCδ nuclear levels [7,8]. The proximal signaling events of how TRAF3 inhibits the activation of noncanonical NF-κB, NF-κB2, have been explicitly elucidated in the literature (Figure 1) [20–24]. It was found that in the absence of stimulation, TRAF3 and TRAF2 assemble a regulatory complex with cIAP1/2 and NIK. Assembly of this complex requires direct binding between TRAF3 and NIK, and also direct association between TRAF2 and cIAP1/2. TRAF3 and TRAF2 heteromeric interaction bring all 4 proteins into the complex. In this complex, the E3 ubiquitin ligase cIAP1/2 induces K48-linked polyubiquitination of NIK and targets NIK for proteasome-mediated degradation, thereby inhibiting NF-κB2 activation. Therefore, in the absence of stimulation, TRAF3 promotes cellular apoptosis in resting B cells by targeting NIK for degradation and inhibiting NF-κB2 activation (Figure 1A) [20–24].

In response to stimulation by the B cell survival factor BAFF or the co-stimulatory ligand CD154, trimerized BAFF receptor (BAFF-R) or CD40 recruits the translocation of cytoplasmic TRAF3 and TRAF2 to the receptor signaling complex in sphingolipid-enriched membrane rafts (Figure 1B). This releases NIK from the TRAF3-TRAF2-cIAP1/2 complex, allowing NIK accumulation, which induces the phosphorylation of IKKα and then the processing of NF-κB2 from the inactive precursor p100 into the active p52. Processed p52 and RelB dimers subsequently translocate into the nucleus to induce the transcription of anti-apoptotic proteins of the Bcl2 family, including Bcl2, Bcl-xL and Mcl1, thereby promoting B cell survival (Figure 1B) [18,25]. Similar to BAFF or CD40 stimulation, deletion of TRAF3 from B cells, caused by either biallelic deletions or inactivating mutations of the *Traf3* gene, also releases NIK from the TRAF2-cIAP1/2 complex and thus allows NIK accumulation, leading to constitutive NF-κB2 activation and BAFF-independent survival of B cells (Figure 1C). However, how TRAF3 promotes PKCδ nuclear translocation remains to be determined.

## Targeting NF-κB2 Activation in TRAF3^−/−^ B Cell Neoplasms

**Figure 2:**
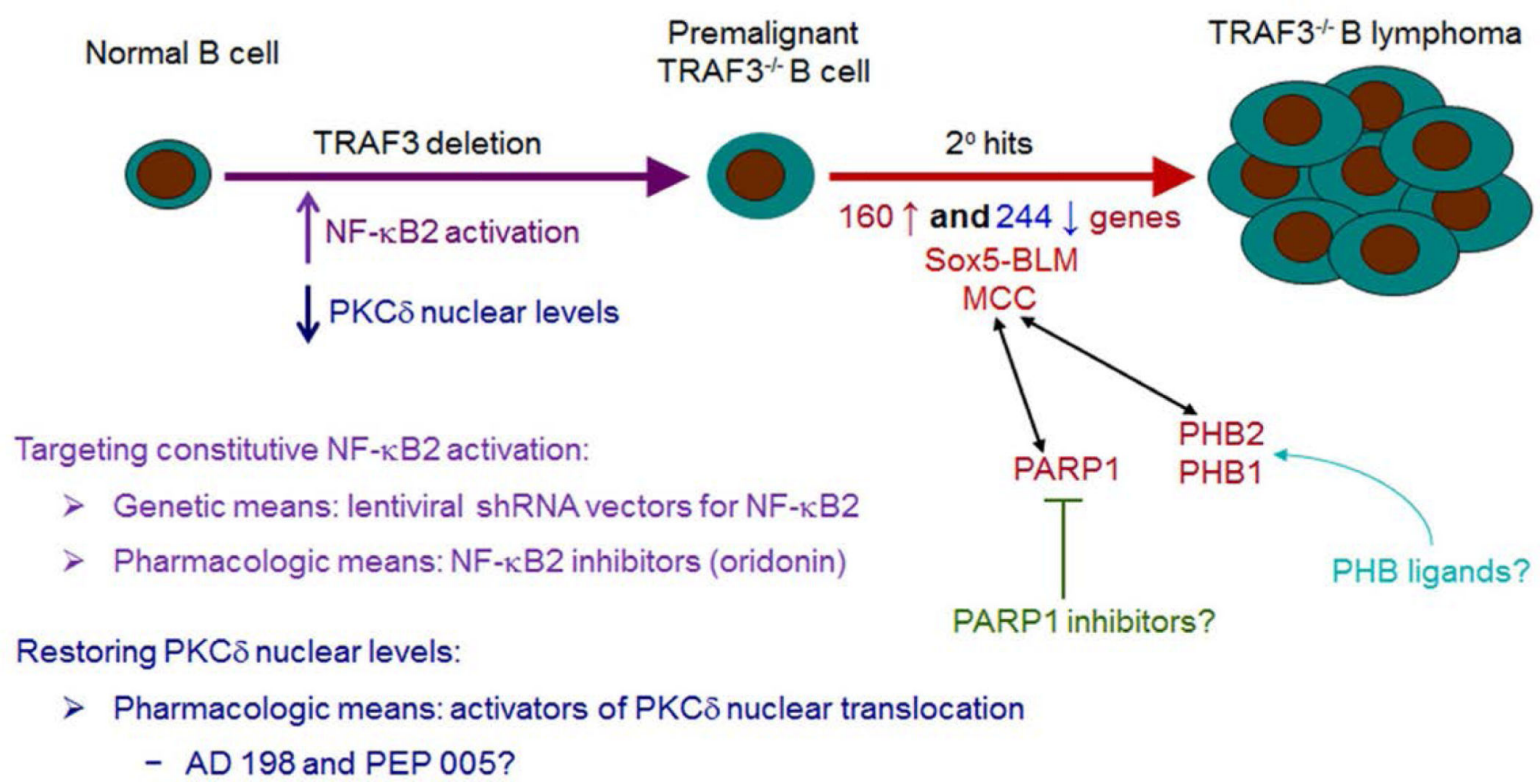
Targeting TRAF3 downstream signaling pathways in B cell neoplasms To test whether constitutive NF-κB2 activation can serve as a therapeutic target in B cell neoplasms, we employed genetic and pharmacological means to target the NF-κB2 pathway. Our results demonstrated that lentiviral shRNA vector-mediated knockdown of NF-κB2 robustly inhibits the proliferation and induces apoptosis in TRAF3^−/−^ B lymphoma cells. We also found that oridonin, an inhibitor of NF-κB2, exhibits potent anti-tumor activities on TRAF3^−/−^ B lymphomas and human MM. To restore PKCδ nuclear levels, we tested two pharmacological activators of PKCδ nuclear translocation, AD 198 and PEP005. We found that AD 198, but not PEP005, potently kills malignant B cells. However, AD 198 does not induce PKCδ nuclear translocation but specifically targets c-Myc in malignant B cells. Therefore, further studies (using genetic means or more specific PKCδ activators) are required to evaluate the therapeutic potential of activating PKCδ nuclear translocation in B cell neoplasms. Interestingly, TRAF3^−/−^ B cells purified from young B-TRAF3^−/−^ mice exhibit prolonged survival but do not proliferate autonomously, and are therefore premalignant B cells. This suggests that additional oncogenic alterations are required for B lymphomagenesis. To identify such secondary oncogenic alterations, we performed a microarray analysis and identified 160 up-regulated genes and 244 down-regulated genes in TRAF3^−/−^ B lymphomas. Among these, we further studied a novel oncogene, *MCC*, and a new isoform of Sox5, Sox5-BLM, expressed in TRAF3^−/−^ B lymphomas. Since our proteomic data identified PARP1 and PHB2/PHB1 as two signaling hubs of MCC in human MM cells, we are currently testing the therapeutic efficacy of PARP1 inhibitors and PHB ligands in B cell neoplasms with TRAF3 inactivation or aberrant *MCC* expression.

Based on the above findings about TRAF3 downstream signaling pathways, we first tested whether constitutive NF-κB2 activation can serve as a therapeutic target in B cell neoplasms with TRAF3 deletions or inactivating mutations. To test this, we employed genetic and pharmacological means to target the NF-κB2 activation (Figure 2). Using lentiviral shRNA vectors, we demonstrated that NF-κB2 shRNA 653 and 1226 knock down both p100 and p52 NF-κB2 proteins by ~95% and ~ 75% reductions, respectively. Interestingly, both NF-κB2 shRNA 653 and 1226 inhibit the proliferation and induce apoptosis in TRAF3^−/−^ mouse B lymphoma cells [8]. Importantly, the potency of the two shRNAs in knocking down NF-κB2 protein levels correlates with their ability in inhibiting the proliferation and inducing apoptosis in TRAF3^−/−^ B lymphoma cells [8]. These data suggest that constitutive NF-κB2 activation is one major oncogenic pathway in TRAF3^−/−^ B cells, which could be therapeutically targeted.

In an effort to test pharmacological means to target the NF-κB2 activation, we examined the effects of an inhibitor of NF-κB2, oridonin, and an inhibitor of IKKβ, BMS-345541, on primary TRAF3^−/−^ B lymphoma cells [8]. The effects of these agents were compared with the activities of drugs used clinically to treat B lymphomas or leukemias, including vincristine, all *trans*-retinoic acid (ATRA), doxorubicin, and cyclophosphamide. We found that oridonin exhibits potent dose-dependent tumoricidal activity on primary TRAF3^−/−^ B lymphoma cells, while BMS-345541 and the four clinical drugs are inactive [8]. We also verified the therapeutic effects of oridonin using three TRAF3^−/−^ B lymphoma cell lines (27-9, 105-8, and 115-6) derived from B-TRAF3^−/−^ mice [8,26] and three human patient-derived MM cell lines (8226, KMS11, and LP1) containing TRAF3 deletions or mutations (Edwards and Xie, unpublished data). Using transplanted TRAF3^−/−^ mouse B lymphoma models, we further demonstrated that oridonin exhibits potent anti-tumor activity in whole animals and is able to prolong the survival of the tumor-bearing mice [26]. Together, our *in vitro* and *in vivo* data provided the preclinical evidence for the therapeutic potential of oridonin in the treatment of B cell neoplasms with TRAF3 deletions or inactivating mutations.

To understand the mechanisms of oridonin, we determined its effects on nuclear levels of NF-κB2 and NF-κB1 subunits in TRAF3^−/−^ B lymphoma cells. Our results showed that oridonin predominantly inhibits the activation of NF-κB2, but also moderately reduces the activation of NF-κB1 in TRAF3^−/−^ B lymphoma cells [8]. In contrast, nuclear translocation of PKCδ, or activation of ERK, p38, JNK, and AKT is unaffected by oridonin [8]. Thus, the potent tumoricidal effects of oridonin can be ascribed to its activity in inhibiting the activation of NF-κB2 (primarily) and also NF-κB1 (moderately). Our findings suggest that oridonin or closely related NF-κB2 inhibitors should be considered as new candidates for the treatment of B cell neoplasms characterized by genetic/epigenetic inactivation of TRAF3 or TRAF3-dependent signaling pathways.

## Targeting PKCδ Nuclear Translocation in TRAF3^−/−^ B Cell Neoplasms

Available evidence suggests that the second signaling pathway downstream of TRAF3 inactivation, the reduced PKCδ nuclear translocation, also contributes to prolonged B cell survival. First, the splenic B cell numbers of PKCδ^−/−^ mice are greatly increased [27,28], similar to that observed in B-TRAF3^−/−^ mice [7,9] and BAFF- or NF-κB2 transgenic mice [29,30]. Second, the principal B cell survival factor, BAFF, also decreases PKCδ nuclear levels in splenic B cells [31]. In light of the above evidence, we next sought to evaluate the therapeutic potential of PKCδ activation in B cell neoplasms with TRAF3 deletions or inactivating mutations (Figure 2). To restore PKCδ nuclear levels, we tested two pharmacological activators of PKCδ nuclear translocation, N-benzyladriamycin-14-valerate (AD 198) and ingenol-3-angelate (PEP005) [32–37]. We found that AD 198 potently kills TRAF3^−/−^ mouse B lymphoma and human MM cell lines with TRAF3 deletions or mutations [26]. In contrast, PEP005 displays contradictory anti- or pro-tumor activities on different malignant B cell lines [26].

Our detailed mechanistic investigation revealed that AD 198 and PEP005 act through distinct biochemical mechanisms in malignant B cells [26]. Interestingly, although PKCδ was identified as the principal target of AD 198 in other cancer cells [32,33], AD 198-induced apoptosis of malignant B cells is mediated through PKCδ-independent mechanisms. We found that AD 198 does not induce PKCδ nuclear translocation in both TRAF3^−/−^ mouse B lymphoma and human MM cells [26]. In contrast, PEP005 promotes the rapid translocation of PKCδ from the cytosol to the nuclei and membranes (including mitochondria) in TRAF3^−/−^ malignant B cells [26]. Interestingly, PEP005 also activates multiple additional signaling pathways in these cells, including PKCα, PKCε, NF-κB1, ERK, JNK, and Akt [26].

In cancer, PKCα and PKCε are generally linked to proliferation or survival and thus considered as oncogenes. In contrast, PKCδ has a pro-apoptotic function in a variety of cancer cells [34,36,38]. Activation of PKC isoforms signals further downstream events, such as the activation of p38, ERK, JNK or NF-κB1 in melanoma, myeloid leukemia and colon cancer cells [37–41], which are all observed in malignant B cells [26]. Therefore, the ultimate effect of cell proliferation or apoptosis induction by PEP005 depends on the balance between the levels and activities of pro-apoptotic (PKCδ) and anti-apoptotic (PKCα and PKCε) isoforms of PKC as well as their crosstalk with different signaling pathways (MAPKs, NF-κB1, and Akt) in each malignant B cell line. Indeed, we detected varying levels of different PKC isoforms (α, δ and ε) in different TRAF3^−/−^ mouse B lymphoma and human MM cell lines, and this may contribute to the observed divergent responses of these cells to PEP005 [26]. Our findings provide new insights into the complexity of the signaling pathways controlled by PEP005 in malignant B cells. However, further studies (using genetic means or more specific PKCδ activators) are required to establish the possibility of activating PKCδ nuclear translocation as a therapeutic avenue for B cell neoplasms with TRAF3 deletions or inactivating mutations.

## AD198 Specifically Targets c-Myc in B Cell Neoplasms

In pursuing the therapeutic mechanisms of AD 198, we found that it specifically targets c-Myc in TRAF3^−/−^ mouse B lymphoma and human MM cells in dose-dependent and time-dependent manner [26]. Both the mRNA and protein levels of c-Myc are drastically and rapidly suppressed by AD 198 [26]. AD 198 inhibits c-Myc protein levels as early as 1 hour after treatment in TRAF3^−/−^ mouse B lymphoma and human MM cell lines [26]. The c-Myc protein is a central regulator of B cell survival and proliferation, and has a short half-life (about 20 – 30 minutes) [42,43]. The promoter regions of both human and mouse *c-Myc* genes contain binding sites for AP-1, a transcription factor directly activated by ERK, p38 and JNK signaling pathways [44–46]. AP-1 is also indirectly inhibited by Akt activity [44]. Interestingly, we found that AD 198 inhibits ERK, p38 and JNK activation, but promotes Akt activation in TRAF3^−/−^ malignant B cells [26]. In this context, our results suggest that AD 198 targets c-Myc by inhibiting c-Myc transcription in malignant B cells, which is mediated through inhibition of ERK, p38 and JNK pathways as well as activation of the Akt pathway [26]. However, we could not exclude additional mechanisms, especially considering that AD 198 inhibits *E. coli* RNA polymerase or chicken leukemic RNA polymerase activity through drug-template interaction or enzyme inactivation, respectively [47]. Regardless of the exact mechanisms, our results showed that AD 198 potently kills TRAF3^−/−^ mouse B lymphoma and human MM cells by targeting c-Myc.

Given that elevated expression of c-Myc is ubiquitously associated with numerous B cell malignancies [48], we extended the investigation of AD 198 to TRAF3-sufficient B lymphoma cell lines. We found that AD198 also potently inhibits the proliferation/survival and suppresses c-Myc expression in six TRAF3-sufficient mouse and human B lymphoma cell lines [26]. Thus, AD 198 also has therapeutic potential and targets c-Myc in TRAF3-sufficient B lymphomas. To further understand whether c-Myc suppression is the therapeutic mechanism of AD 198 in malignant B cells, we performed c-Myc reconstitution experiments. We generated a lentiviral expression vector of FLAG-tagged human c-Myc, pUB-FLAG-c-Myc-Thy1.1, in which constitutive expression of FLAG-c-Myc is driven by the ubiquitin promoter (pUB). Following treatment with AD 198, although endogenous c-Myc protein levels are strikingly decreased, the transduced FLAG-c-Myc protein levels are not suppressed by AD 198 in human MM cells [26]. The observation that expression of the transduced FLAG-c-Myc (driven by the ubiquitin promoter) is not suppressed by AD 198 indicates that AD 198 targets the transcription of endogenous c-Myc via its effects on the c-Myc promoter. In support of the major role of c-Myc down-regulation, we found that lentiviral vector-mediated constitutive expression of FLAG-c-Myc confers robust resistance to the anti-tumor effects of AD 198 in human MM cells [26]. Together, our results indicate that c-Myc suppression is a major contributing factor to the anti-tumor effects of AD 198 in both TRAF3^−/−^ and TRAF3-sufficient B cell neoplasms.

Using transplanted TRAF3^−/−^ mouse B lymphoma models, we also demonstrated that AD 198 has potent anti-tumor activities in whole animals. It has been previously shown that AD 198 does not exhibit significant organ toxicities at therapeutic doses, and is cardioprotective in rodent models [40,49–51]. Indeed, we found that in NOD SCID mice transplanted with TRAF3^−/−^ mouse B lymphomas, administration of AD 198 drastically extends the survival of mice and inhibits the growth and metastasis of B lymphomas [26]. In fact, AD 198 demonstrates a higher *in vivo* potency than oridonin, an inhibitor of NF-κB2 and NF-κB1 pathways [26]. Our findings thus support further clinical studies of AD 198 as an anti-cancer agent for B cell neoplasms involving TRAF3 inactivation or Myc up-regulation.

## Secondary Oncogenic Hits in TRAF3^−/−^ Mouse B Lymphomas

We noticed that TRAF3^−/−^ B cells purified from young B-TRAF3^−/−^ mice exhibit prolonged survival but do not proliferate autonomously [7], and are therefore premalignant B cells. Consistent with this, no B lymphoma development is observed in B-TRAF3^−/−^ mice younger than 9 months old [8]. The long latency of B lymphoma development observed in B-TRAF3^−/−^ mice suggests that TRAF3 inactivation and its downstream signaling pathways are not sufficient and that additional oncogenic alterations are required for B lymphomagenesis (Figure 2). Although TRAF3 deletions or mutations exist in human patients with B cell neoplasms, it is not known whether TRAF3 inactivation is the primary or secondary oncogenic event in human samples. Therefore, B-TRAF3^−/−^ mice offer the unique advantage to identify secondary oncogenic pathways that drive B lymphomagenesis in the context of TRAF3 inactivation.

To identify such secondary oncogenic alterations and to discover new therapeutic targets for the treatment of B cell neoplasms, we performed global gene expression profiling of TRAF3^−/−^ mouse B lymphomas by transcriptomic microarray analysis [52,53]. Our results of the microarray analysis identified 160 up-regulated genes and 244 down-regulated genes in TRAF3^−/−^ B lymphomas as compared to TRAF3-sufficient littermate control spleens (cut-off fold of changes: 2-fold up or down, *p* < 0.05) (NCBI GEO accession number: GSE48818) [52,53]. From the 160 up-regulated genes, we selected 13 genes for further verification by quantitative real time PCR using TaqMan gene expression assay kits. Our data verified the mRNA up-regulation of the 13 genes examined, including *MCC*, *Sox5*, *Diras2*, *Tbc1d9*, *Ccbp2*, *Btbd14a*, *Sema7a*, *Twsg1*, *Ppap2b*, *TCF4*, *Tnfrsf19*, *Zcwpw1*, and *Abca3* [52,54]. Striking up-regulation of these transcripts was verified in the three splenic B lymphoma samples used for the microarray analysis, and also confirmed in three additional splenic B lymphomas as well as ascites from two cases [52,54]. Thus, these 13 genes are recurrently up-regulated in B lymphomas spontaneously developed in different individual B-TRAF3^−/−^ mice, and need to be further investigated as candidates of diagnostic markers or therapeutic targets in B cell neoplasms. We have further studied two of the up-regulated genes, *MCC* and *Sox5*.

## *MCC*: A Novel Oncogene in B Lymphocytes

*Mutated in colorectal cancer* (*MCC*) was originally identified as a tumor suppressor gene in colorectal cancer (CRC). Our unexpected finding that MCC is strikingly up-regulated in TRAF3^−/−^ mouse B lymphomas prompted us to further investigate the expression and function of MCC in B cell neoplasms. We demonstrated high levels of aberrant *MCC* expression in six human MM cell lines with TRAF3 deletions or relevant mutations [52]. *MCC* expression is also significantly elevated in a variety of primary human B cell malignancies, including primary effusion lymphoma (PEL), centroblastic lymphoma (CBL), diffuse large B-cell lymphoma (DLBCL), Burkitt’s lymphoma (BL), and MM [55–58]. However, expression of the transcript and protein of *MCC* is not detected in normal B cells or premalignant TRAF3^−/−^ B cells, even after treatment with a variety of B cell stimuli, including CD40, B cell receptor (BCR), LPS (a TLR4 agonist), and CpG (a TLR9 agonist) [52]. These results suggest that aberrant *MCC* expression is specifically associated with B cell neoplasms.

We next investigated the functions of MCC in malignant B cells. In contrast to the cell cycle blocking and proliferation inhibitory effects of MCC overexpression reported in fibroblasts and CRCs [59–62], we observed that overexpression of MCC does not affect cell cycle progression, cell proliferation, or cell survival in human MM cells [52]. These results thus argue against a negative role for MCC in the survival or proliferation of malignant B cells. Furthermore, we found that lentiviral shRNA vector-mediated knockdown of MCC induces apoptosis and inhibits proliferation in human MM cell lines with TRAF3 deletions or mutations [52]. Interestingly, MCC shRNA 1332 that results in a greater decrease in MCC protein level is also most effective at inducing apoptosis and inhibiting proliferation in human MM cells [52]. Together, our results demonstrate that MCC plays a positive role and is required for the survival and proliferation in human MM cells, indicating that *MCC* acts as an oncogene in B lymphocytes. Our knockdown studies also suggest that MCC could serve as a therapeutic target in B cell neoplasms.

To elucidate where MCC exerts its oncogenic roles in malignant B cells, we examined the subcellular localization of MCC using a biochemical fractionation method. Our results revealed that MCC protein is primarily localized in mitochondria, but also detectable in the ER, cytosol and nucleus in human MM cells [52]. To understand the mechanisms of MCC, we investigated MCC downstream signaling pathways using complementary overexpression and knockdown approaches. We found that MCC inhibits cleavage of caspases 8 and 3, down-regulates the cell cycle inhibitor p27, and up-regulates Mcl1, c-Myc and cyclin B1 as well as ERK phosphorylation in human MM cells [52]. Furthermore, we identified 365 proteins of the MCC-interactome in human MM cells using affinity purification followed by LC-MS/MS [52]. Among these, PARP1 and prohibitin-2 (PHB2) were recognized as two signaling hubs of the MCC interaction network in human MM cells [52]. PARP1 and PHB2/PHB1 have been previously shown to directly or indirectly interact with and/or regulate all MCC downstream targets identified in our study, including ERK, c-Myc, p27, cyclin B1, Mcl-1, caspase 8, and caspase 3 [63–66]. Taken together, our results indicate that MCC promotes cellular survival and proliferation by interacting with and modulating the interaction network centered at PARP1 and PHB2/PHB1 in malignant B cells. Given the preclinical evidence that both PARP1 and PHB2/PHB1 are excellent therapeutic targets in human cancers [63,66], our findings also implicate potential applications of PARP inhibitors and PHB ligands in the treatment of B cell neoplasms involving TRAF3 inactivation or aberrant *MCC* expression (Figure 2).

## A Novel Isoform of Sox5, Sox5-BLM, is Expressed in TRAF3^−/−^ B Lymphomas

Sox5 is a member of the Sox family of transcription factors. Corroborating our finding that Sox5 is strikingly up-regulated in TRAF3^−/−^ mouse B lymphomas [54], Sox5 up-regulation has also been documented in DLBCL developed in Brd2 transgenic mice (GEO accession number: GSE6136) [67] and MM developed in XBP-1 transgenic mice (GEO accession number: GSE6980) [68]. Importantly, up-regulation of SOX5 mRNA has also been identified in human memory B cells [69], in clonal B cells of patients with hepatitis C virus (HCV)-associated B cell lymphoproliferative disorders mixed cryoglobulinemia [70], and in human follicular lymphoma (FL) [71,72] and hairy cell leukemia (a sub-type of chronic lymphoid leukemia of B lymphocytes) [55]. Interestingly, up-regulation of SOX5 expression in a case of human FL is caused by a chromosomal translocation t(X;12), which fuses the promoter region of the G-protein coupled purinergic receptor *P2Y8* gene with the *SOX5* coding sequence [71]. In contrast, Sox5 protein expression is not detectable in normal B cells or premalignant TRAF3^−/−^ B cells even after treatment with B cell stimuli [54], suggesting that up-regulation of Sox5 protein is selectively associated with B cell malignant transformation.

Our detailed cloning and sequencing studies revealed that the Sox5 expressed in TRAF3^−/−^ mouse B lymphomas represents a novel isoform of Sox5, Sox5-BLM (GenBank accession number: KF793916), which contains a 35 amino acid (aa) deletion in the N-terminal region in front of the leucine zipper domain [54]. Our biochemical fractionation results demonstrated that Sox5-BLM is primarily localized in the nucleus of malignant B cells [54]. Furthermore, we found that Sox5-BLM regulates cell cycle progression by modulating p27 and β-catenin protein levels in transduced human MM cells [54]. However, whether SOX5 can serve as a therapeutic target in human B cell neoplasms involving TRAF3 inactivation or aberrant *SOX5* expression awaits further investigation.

## Conclusions

In summary, our study identified a variety of candidate diagnostic markers for B cell malignancies, including TRAF3 inactivation, NF-κB2 constitutive activation, reduced PKCδ nuclear levels, and aberrant expression of MCC or SOX5. Our study also discovered a number of candidate therapeutic targets for the treatment of B cell neoplasms, including NF-κB2, PKCδ, MCC, PARP1, and PHB2. We have tested several drugs targeting NF-κB2 and PKCδ, and found that both oridonin and AD198 exhibit potent anti-tumor activities on B cell neoplasms with TRAF3 deletions or inactivating mutations. Oridonin acts by inhibiting the activation of NF-κB2 (primarily) and also NF-κB1 (moderately). Although we originally tested AD 198 as an activator of PKCδ, our results revealed that AD 198 does not affect PKCδ but specifically targets c-Myc in malignant B cells. Since our new proteomic data identified PARP1 and PHB2/PHB1 as two signaling hubs of the novel oncoprotein MCC in human MM cells, we are currently testing the therapeutic efficacy of PARP1 inhibitors and PHB ligands in B cell neoplasms with TRAF3 inactivation or aberrant *MCC* expression. Collectively, our findings indicate that restoration of TRAF3 downstream signaling pathways represents an important line of new therapeutic avenues for B cell neoplasms.

## Abbreviations

AD 198: N-benzyladriamycin-14-valerate
BAFF: B Cell Activating Factor
BAFF-R: BAFF Receptor
B-CLL: B Cell Chronic Lymphocytic Leukemia
BCR: B Cell Receptor
BL: Burkitt’s Lymphoma
B-TRAF3^−/−^: B Cell-specific TRAF3-deficient
CBL: Centroblastic Lymphoma
cIAP1/2: Cellular Inhibitor of Apoptosis Proteins ½
CRC: Colorectal Cancer
DLBCL: Diffuse Large B-cell Lymphoma
ER: Endoplasmic Reticulum
ERK: Extracellular Signal-regulated Kinase
JNK: c-Jun N-terminal Kinase
LPS: Lipopolysaccharides
LC-MS/MS: Liquid Chromatography-Mass Spectrometry/Mass Spectrometry
MCC: Mutated in Colorectal Cancer
MCL: Mantle Cell Lymphoma
MM: Multiple Myeloma
MZL: Splenic Marginal Zone Lymphoma
NF-κB: Nuclear Factor κ Light Chain Enhancer of Activated B Cells
NIK: NF-κB Inducing Kinase
PARP1: Poly [ADP-ribose] Polymerase 1
PEL: Primary Effusion Lymphoma
PEP005: Ingenol-3-angelate
PHB2: Prohibitin-2
shRNA: Short Hairpin RNA
TRAF3: Tumor Necrosis Factor Receptor (TNF-R)-associated Factor 3
WM: Waldenström’s Macroglobulinemia
